# *In vivo* and *in vitro* protective effect of arginine against intestinal inflammatory response induced by *Clostridium perfringens* in broiler chickens

**DOI:** 10.1186/s40104-019-0371-4

**Published:** 2019-08-12

**Authors:** Beibei Zhang, Liping Gan, Muhammad Suhaib Shahid, Zengpeng Lv, Hao Fan, Dan Liu, Yuming Guo

**Affiliations:** State Key Laboratory of Animal Nutrition, College of Animal Science and Technology, China Agricultural University, Beijing, 100193 People’s Republic of China

**Keywords:** Arginine, Broiler chickens, *Clostridium perfringens*, Intestine inflammatory response, Metabolism

## Abstract

**Background:**

Necrotic enteritis is a widespread disease in poultry caused by *Clostridium perfringens.* We previously reported that dietary arginine supplementation protected the intestinal mucosa of broiler chickens with necrotic enteritis, but the related protective mechanisms remain unclear. The *in vivo* trial was designed as a 2 × 2 factorial arrangement to evaluated the effects of arginine supplementation on inflammatory responses, arginine transporters, arginine catabolism and JAK-STAT signalling pathway in broiler chickens challenged with *C. perfringens* or without *C. perfringens*. Furthermore, we validated the *in vivo* results using intestinal epithelial cells of chicken embryos.

**Results:**

*C. perfringens* infection markedly increased gut gross pathological and histopathological lesion scores, promoted liver *C. perfringens* invasion, reduced serum arginine levels, and elevated jejunal mucosal lysozyme activities (*P* < 0.05), but these effects were significantly reversed by arginine supplementation *in vivo* (*P* < 0.05). The challenge significantly increased serum procalcitonin levels, jejunal mucosal iNOS activities and jejunal *IL-6*, *TGF-β3*, cationic amino acid transporter (*CAT*)*-1*, and *CAT-3* mRNA expression (*P* < 0.05), whereas arginine supplementation significantly reduced jejunal *IFN-γ*, *IL-1β*, *IL-6*, *IL-10*, *TGF-β3*, and *CAT-3* mRNA expression (*P* < 0.05). Arginine supplementation significantly attenuated the *C. perfringens* challenge-induced increases in jejunal *iNOS*, arginase 2, arginine decarboxylase, arginine:glycine amidinotransferase, *JAK1*, *JAK3*, *STAT1*, and *STAT6* mRNA expression (*P* < 0.05). The *in vitro* experiment showed that *C. perfringens* challenge markedly increased cellular cytotoxicity and the mRNA expression of *IL-1β*, *IL-8*, *IL-10*, *CAT-1* and *CAT-3* (*P* < 0.05), which were significantly reversed by 50 μmol/L and/or 400 μmol/L arginine pre-treatment (*P* < 0.05).

**Conclusions:**

Arginine prevented *C. perfringens* challenge-induced circulated arginine deficiency, normalized intestinal arginine transport and catabolism, down-regulated JAK-STAT signalling pathway and attenuated the inflammatory response, which exerted protective effects on the intestine of broiler chickens.

## Introduction

Necrotic enteritis is a widespread avian intestinal disease which causes significant economic loss in the poultry industry [1]. In the clinical cases, this leads to depression, dehydration, diarrhoea, decreased feed intake and acute death while in subclinical cases, it generates chronic intestinal mucosal damage, poor growth performance without mortality [2, 3]. It is certain that the overgrowth of *C. perfringens* and its extracellular toxins are the main causative agent of necrotic enteritis [2, 3]. However, several predisposing factors may be required to elicit the clinical signs and lesions of necrotic enteritis, such as coccidiosis, immunosuppressive agents, diets containing a high level of non-starch polysaccharides (wheat, rye, and barley), and a novel pore-forming toxin, NetB [3]. Due to the restrictions of antibiotic growth promoters in a growing number of countries and regions, the high prevalence of necrotic enteritis is prompting an active search for dietary strategies to prevent its occurrence or control its development [2].

*L*-arginine is an essential amino acid for chickens and turns out to be a conditionally essential amino acid for mammals under many stressful conditions [4–6]. The intestine is an important organ for maintaining body arginine homoeostasis [7] as arginine is actively transported and metabolized by enterocytes. Cellular arginine uptake is mainly mediated by the cationic amino acid transporter (CAT) family, also known as y^+^ transporters, which is involved in active transport [8]. Arginine is catabolized via multiple pathways mediated by nitric oxide synthase, arginase, arginine:glycine aminotransferase (AGAT), and arginine decarboxylase (ADC) [9]. The alteration of arginine uptake and metabolism has been found to be strongly related to various enteric diseases, such as ulcerative colitis [10] and *Citrobacter rodentium* colitis [11]. It has been reported that infection-associated arginine deficiency contributes to intestinal immunopathology, and arginine administration can significantly attenuate intestinal inflammation and infectious complications [12]. Chau et al. [13] demonstrated that malaria-associated hyperargininaemia could impair intestinal barrier functions, rendering the host vulnerable to *Salmonella* coinfection. Increasing arginine bioavailability through oral supplementation can ameliorate intestinal inflammation and pathological lesions. In a *Citrobacter rodentium*-induced colitis model, serum arginine levels of the infected mice were significantly lower than those of the uninfected mice, and arginine supplementation was clinically beneficial [11]. Thus, dietary arginine administration may be a necessary strategy for maintaining arginine homeostasis under many physiological and pathological conditions to maintain good health and bodily functions [14].

The Janus kinase (JAK)-signal transducer and activator of transcription (STAT) cascade is a critical signaling pathway in inflammatory responses [15]. It mediates the transcription of inflammatory cytokines, including *IL-6*, *IL-1β* and *TNF-α*, and inducible enzymes such as cyclooxygenase-2 and inducible nitric oxide synthase (*iNOS*) in mammals [16, 17]. Truong et al. [18] demonstrated that mRNA expression of *JAK1*, *JAK2*, *JAK3*, and *STAT1* in the spleen of broilers suffering from necrotic enteritis were dramatically elevated compared with that of healthy birds. However, little is known about the role of the JAK-STAT signalling pathway in the protective effect of arginine supplementation against necrotic enteritis in broiler chickens.

We previously demonstrated that dietary arginine supplementation protected the intestinal mucosa of broiler chickens with necrotic enteritis by promoting innate immune responses, improving intestinal absorption and barrier function, and inhibiting *C. perfringens* colonization [19]. However, the effects of dietary arginine supplementation on intestinal arginine transport, metabolism and the related protective mechanism in broilers with necrotic enteritis remain uncertain. Thus, the present study was designed to determine the effects of arginine supplementation on inflammatory responses induced by *C. perfringens* *in vivo* and *in vitro*, and explore the associated, potentially protective mechanisms.

## Materials and methods

### *In vivo* experiments

#### Animals and experimental treatments

In a 2 × 2 factorial arrangement, 140 one-day-old male Arbor Acres broiler chickens were randomly allocated to four groups, with 35 birds per group. The treatments were as follows: CON group, received a basal diet; ARG group, fed a basal diet supplemented with 3 g/kg arginine; CON+CP group, received a basal diet and underwent *C. perfringens* challenge; and ARG + CP group, given a basal diet supplemented with 3 g/kg arginine and underwent *C. perfringens* challenge. The basal diet was formulated according to the recommendations by the National Research Council (NRC 1994), and alanine was used to keep crude protein level constant for different diets (Table 1). High-performance liquid chromatography (HPLC) was used to determine the dietary amino acid levels. Each group of birds was kept in a separate rearing isolator (1.6 m × 0.7 m × 0.7 m). Each isolator contains a wire mesh floor and was equipped with two nipple drinkers and one feeder. Air was pumped into the isolator and the room housed with the isolators was air-conditioned. The birds were maintained on a 23 h/d-lighting programme and routinely immunized. The experiment lasted for 21 d.

**Table 1:** Diet composition and nutrient levels

Items	Control diet	l-arginine-supplemented diet
Ingredient, g/kg (unless otherwise indicated)		
Corn	582.8	582.8
Soybean meal, 44% CP	291.1	291.1
Corn gluten meal	50.0	50.0
Soybean oil	25.0	25.0
Dicalcium phosphate	19.2	19.2
Limestone	8.9	8.9
Sodium chloride	3.0	3.0
Choline chloride, 50%	2.5	2.5
Mineral premix^a^	2.0	2.0
d,l-Methionine, 98%	2.6	2.6
l-Lys-HCl, 98%	2.4	2.4
Vitamin premix^b^	0.3	0.3
Ethoxyquin, 33%	0.2	0.2
l-Arg	0.0	4.0
l-Ala	10.0	6.0
Nutrient level^c^, g/kg (unless otherwise indicated)		
ME, Mcal/kg	2.98	2.98
CP	219.4	219.4
Ca	10.0	10.0
Non-phytate phosphorus	4.5	4.5
Lys, measured value	13.6	13.9
Met	5.9	5.9
Thr, measured value	8.6	8.8
Arg, measured value	14.2	17.2

^a^Supplied per kilogram of complete feed: Mn, 100 mg; Fe, 80 mg; Zn, 75 mg; Cu, 8 mg; I, 0.35 mg; and Se, 0.15 mg

^b^Supplied per kilogram of complete feed: vitamin A, 12,500 IU; vitamin D_3_, 2500 IU; vitamin E, 30 IU; vitamin K_3_, 2.65 mg; vitamin B_1_, 2 mg; vitamin B_2_, 6 mg; vitamin B_5_, 12 mg; vitamin B_12_, 0.025 mg; niacin, 50 mg; folic acid, 1.25 mg and biotin, 0.0325 mg

^c^Calculated composition, unless otherwise indicated

#### *C. perfringens* challenge

A chicken *C. perfringens* type A strain (CVCC2030, China Veterinary Culture Collection Center, Beijing, China) was used to establish a necrotic enteritis model based on the method of a previous study [20]. Briefly, the *C. perfringens* strain was cultured anaerobically in a broth culture medium containing beef pellets overnight at 37 °C, and the viable bacteria were counted on tryptose-sulfite-cycloserine agar (CM138, Land Bridge Technology Ltd., Beijing, China) plates with d-cycloserine supplement (P-15B, Land Bridge Technology Ltd., Beijing, China). From d 14 to d 20, 1 mL (2~3) × 10^8^ CFU/mL) of fresh *C. perfringens* culture was orally administered to all birds in the challenged groups (group CON+CP and ARG + CP) per day, while birds in the non-challenged groups (group CON and ARG) received equal volumes of sterile broth culture medium.

#### Sample collection

On d 21, eight birds from each group were randomly selected, and blood samples were individually collected from the wing vein and then were centrifuged at 3,000 r/min for 10 min at 4 °C to obtain serum for determining arginine and procalcitonin concentrations. Then, the chickens were killed by intravenous injection of pentobarbital sodium (30 mg/kg body weight) and jugular exsanguination. The midregion of the jejunum (~ 1 cm) was sampled, flushed gently with saline to remove digesta and immediately frozen in liquid nitrogen, and stored at − 80 °C for subsequent RNA extraction. Mucosa from the second half of the jejunum (from the midpoint of jejunum to Meckel’s diverticulum) was scrapped, immediately frozen in liquid nitrogen and stored at − 20 °C for subsequent determination of lysozyme and iNOS activities.

#### Intestinal lesion score and histopathological examination

The gross macroscopic lesions were scored according to the method of Dahiya et al. [21] with some modifications. The small intestine from each bird was firstly observed from the serosal side, then opened longitudinally and last observed from the mucosal side. The lesion scores system was as follows: 0 = normal appearance, no lesion; 0.5 = severely congested serosa and mesentery engorged with blood; 1 = thin-walled and friable intestine with small red petechiae (> 5); 2 = focal necrosis and small amounts of gas production; 3 = patches of necrosis (1 to 2 cm long) and gas-filled intestine; and 4 = diffused necrosis and large amounts of gas. For histopathological examination, jejuna of experimental birds were preserved in 4% paraformaldehyde prior to being dehydrated, embedded in paraffin, sectioned, and stained with hematoxylin and eosin. Histopathological scores were determined according to the method of Jerzsele et al. [22] with some modifications and the scoring criteria were as follows: (i) epithelial cell defects, (ii) lympho-histiocytic infiltration, (iii) villus fusion, (iv) capillary dilation, (v) capillary hemorrhages, (vi) erosion of the epithelial layer. Each attribute was scored using a scale from 0 to 3 (0, none; 1, mild; 2, moderate and 3, severe), and then these were summarized and total scores were attached to each sample. Both gross macroscopic lesions and microscopic histopathological evaluation were scored in a blinded fashion.

#### Liver *C. perfringens* enumerations

The livers from 8 birds per treatment were collected for *C. perfringens* enumerations by plate count. Approximately 0.3 g of each liver sample was weighted, aseptically collected into 5-mL sterile centrifuge tubes, diluted to 1:10 with sterile normal saline and then homogenized using a high-throughput tissue homogenizer (Scientz, Ningbo, China). The liver homogenate was serially diluted with sterile normal saline from 10^− 1^ to 10^− 2^, and then 100 μL of each sample diluent was plated on tryptose-sulfite-cycloseine agar with d-cycloserine supplement for *C. perfringens* enumeration in duplicate. The *C. perfringens* was counted after anaerobic incubation for 24 h at 37 °C. The result was expressed as colony forming units (CFUs) per gram of liver.

#### Serum procalcitonin determination

Serum procalcitonin levels were measured using a chicken procalcitonin ELISA kit (Beijing Jinhai Keyu Biotech Development Co., Ltd., Beijing, China) according to the manufacturer’s guidelines.

#### Serum arginine concentration assay

Serum samples (40 μL) were transferred to 1.5-mL centrifuge tubes, with 40 μL of HClO_4_ (1.5 mol/L) added to remove proteins and 20 μL of K_2_CO_3_ solution added to neutralize the pH. The mixture was diluted with 900 μL of HPLC-grade water and centrifuged at 10,000×*g* for 10 min to precipitate the pellet. The resultant supernatant was used to analyze the arginine concentration following the method of Wu and Meininger [23].

#### Assay for lysozyme and iNOS activities in the jejunal mucosa

The jejunal mucosa (0.3 g) was added to 2.7 mL of sterile saline, homogenized, and centrifuged at 3,000 r/min for 10 min at 4 °C. The supernatant was assayed for lysozyme and iNOS activities using the Lysozyme Assay Kit and the Inducible Nitric Oxide Synthase Assay Kit, respectively, from Nanjing Jiancheng Bioengineering Institute (Nanjing, China) following the manufacturer’s protocols. The results are expressed as units/mg of mucosal protein. The concentration of protein was determined using the BCA Assay Kit (CWBio, Inc., Beijing, China).

#### Jejunal gene expression assay

RNA extraction and real-time PCR were performed as described in a previous study from our lab [24]. Briefly, total RNA was isolated from the jejunal tissues using Trizol reagent (Invitrogen Life Technologies, Carlsbad, California). cDNA was generated using the PrimeScript RT reagent kit with cDNA eraser (Takara, Dalian, China). Quantitative PCR assays were carried out using the SYBR® Premix Ex Taq™ kit (Takara, Dalian, China). Target gene expression was quantified using the 2^-△△CT^ method [25] and normalized to the expression of GAPDH. The PCR primer sequences are shown in Table 2.

**Table 2:** Primers used for real-time PCR analysis^a^

Gene name	Primer sequence (5′ to 3´)	Product size, bp	Efficiency, %	Accession number
*CAT-1*	F: ATGTAGGTTGGGATGGAGCC	280	113.556	XM_015277949.1
	R: AACGAGTAAGCCAGGAGGGT			
*CAT-2*	F: CAAGTCTTCTCGGCTCTAT	105	105.602	XM_015285435.1
	R: GTGCCTGCCTCTTACTCA			
*CAT-3*	F: CAAGACTGGCTCTGCCTACC	236	106.623	XM_015278426.1
	R: GGATCAACGCAAAGAAGTCC			
*iNOS*	F: TGGGTGGAAGCCGAAATA	241	103.087	NM_204961.1
	R: GTACCAGCCGTTGAAAGGAC			
*ARG2*	F: GCCAACTGTACGACTTTGGAG	150	90.430	NM_001199704.1
	R: AGCTGTGTCCAGCAGCTACC			
*ADC*	F: CCCAGTTTGAGGAGATTGCT	122	108.230	XM_004947849.2
	R: AAAGGTGAAGGCTGAGGTGA			
*AGAT*	F: TCGTCAAGAGGCCTGATCCA	140	111.489	XM_015291975.1
	R: CCAAGCCATAGGCGCTTCAA			
*IL-1β*	F: ACTGGGCATCAAGGGCTA	131	104.419	XM_015297469.1
	R: GGTAGAAGATGAAGCGGGTC			
*IL-6*	F: CGCCCAGAAATCCCTCCTC	152	101.974	XM_015281283.1
	R: AGGCACTGAAACTCCTGGTC			
*IL-8*	F: ATGAACGGCAAGCTTGGAGCTG	233	119.885	NM_205498.1
	R: TCCAAGCACACCTCTCTTCCATCC			
*IFN-γ*	F: AGCTGACGGTGGACCTATTATT	259	108.556	NM_205149.1
	R: GGCTTTGCGCTGGATTC			
*IL-10*	F: CGCTGTCACCGCTTCTTCA	88	98.435	NM_001004414.2
	R: TCCCGTTCTCATCCATCTTCTC			
*TGF-β3*	F: CATCGAGCTCTTCCAGATCC	112	102.986	NM_205454.1
	R: GACATCGAAGGACAGCCACT			
*JAK1*	F: TGCACCGTGACTTAGCAGCAAG	168	114.087	XM_015290965.1
	R: TCTGAATCAAGCATTCTGGAGCATACC			
*JAK2*	F: TCGCTATGGCATTATTCG	197	110.029	XM_015280061.1
	R: GTGGGGTTTGGTCCTTTT			
*JAK3*	F: GCATCCGCCGCCGTGTTG	108	96.164	NM_204996.3
	R: AGCACCGCAGCCTCTCCAG			
*STAT1*	F: TAAAGAGGGAGCAATCAC	112	109.954	XM_015289392.1
	R: ATCAGGGAAAGTAACAGC			
*STAT6*	F: GCAACCTCTACCCCAACA	127	101.256	XM_015274736.1
	R: TCCCTTTCGCTTTCCACT			
*GAPDH*	F: TGCTGCCCAGAACATCATCC	142	98.200	NM_204305.1
	R: ACGGCAGGTCAGGTCAACAA			

^a^*Abbreviations*: *CAT* Cationic amino acid transporter, *iNOS* inducible nitric oxide synthase, *ARG2* Arginase 2, *ADC* Arginine decarboxylase, *AGAT* Arginine: glycine amidinotransferase, *IFN-γ* Interferon-γ, *TGF-β3* Transforming growth factor-β3, *JAK* Janus kinase, *STAT* Signal transducer and activator of transcription, *GAPDH* Glyceraldehyde-3-phosphate dehydrogenase, *F* Forward, *R* Reverse

### *In vitro* experiments

#### Cell culture and treatment

The isolation procedure of primary intestinal epithelial cells (IEC) from chicken embryos were performed as described previously [26]. Chicken IEC were grown in Dulbecco’s Modified Eagles Medium/Nutrient Mixture F-12 (DMEM/F12) medium (Macgene technology, Beijing, China) containing 2.5% fetal bovine serum (Gibco, Carlsbad, CA), 1% penicillin-streptomycin (Macgene technology, Beijing, China), 100 μg/mL heparin sodium salt, 5 μg/mL insulin and 20 ng/mL epidermal growth factor (BD Biosciences, San Jose, CA). All other chemicals, unless indicated, were purchased from Sigma-Aldrich (St. Louis, MO). Isolated cells (5 × 10^6^) were seeded in 6-well culture plates (Corning Life Science, Tewksbury, MA) with a total volume of 2 mL of DMEM/F12. After 48 h of incubation, the cells were starved for 8 h in custom-made arginine-free DMEM/F12 (Merck Millipore Beijing Skywing, Beijing, China) containing 1% penicillin-streptomycin. Then they were washed with pre-washed phosphate-buffered saline (PBS) twice and allocated into 4 groups: group 1, cultured in arginine-free media without *C. perfringens* challenge; group 2, cultured in arginine-free media prior to *C. perfringens* challenge; group 3, cultured in 50 μmol/L arginine prior to *C. perfringens* challenge; group 4, cultured in 400 μmol/L arginine prior to *C. perfringens* challenge. After a 6-h period of culture in custom-made DMEM/F12 containing 0 μmol/L, 50 μmol/L or 400 μmol/L *L*-arginine, the cells were washed with PBS twice and then cultured for 4 h in 2 mL arginine-free DMEM/F12 containing *C. perfringens* at multiplicity of infection (MOI, the ratio of bacteria to eukaryotic cells) of 1:1 or equal volume PBS. Following 4 h of incubation, media were collected, centrifuged at 4,000×*g* for 10 min to obtain supernatant for analyzing lactic dehydrogenase (LDH) release, and cells were collected for RNA extraction. Each treatment included 6 wells.

#### Cytotoxicity measurement

LDH, a cytoplasmic enzyme, is quickly released into the extracellular environment when the plasma membrane of a cell is injured [27]. In the *in vitro* experiment, cytotoxicity was assessed by the levels of LDH in the cell supernatant using the LDH cytotoxicity assay kit (Beyotime Biotechnology, Beijing, China).

#### Cellular RNA isolation and gene expression

Total RNA of chicken IEC was extracted and gene expression was measured by RT-PCR as described above.

### Statistical analysis

Data were analyzed using a two-factor ANOVA of a general linear model for the *in vivo* experiment and one-way ANOVA for the *in vitro* experiment using SPSS version 18.0. A univariate ANOVA and Duncan’s multiple comparisons were carried out for significant interactive effects *in vivo*. Differences were considered statistically significant at *P* ≤ 0.05. Data are presented as means ± SEMs.

## Results

### Intestinal gross pathological and histopathological examination, and liver *C. perfringens* invasion *in vivo*

**Fig. 1:**
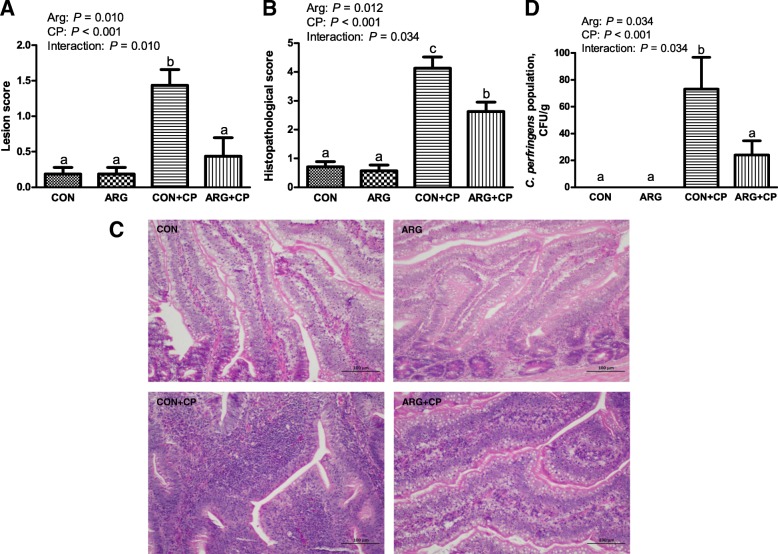
Intestinal injury examination and liver *C. perfringens* number of broiler chickens. **a**: Gross lesion score of the small intestine. **b** and **c**: Histopathological injury score and representative histopathological pictures of the jejunum. The jejunal cross-sections were hematoxylin and eosin-stained. Original magnification is 200×. **d**: Number of *C. perfringens* in the liver. CON group, received a basal diet; ARG group, fed a basal diet supplemented with 3 g/kg arginine; CON+CP group, received a basal diet and underwent *C. perfringens* challenge; ARG + CP group, given a basal diet supplemented with 3 g/kg arginine and underwent *C. perfringens* challenge. The results are expressed as means ± SEM (*n* = 8). Different letters indicate significant differences for the interaction effect (*P* ≤ 0.05)

Most of the birds with only *C. perfringens* challenge showed depression, diarrhea, and inappetence. Furthermore, one bird died in CON+CP group, as well as ARG + CP group, in which necrotic enteritis was confirmed pathologically. There was no mortality in the unchallenged groups. A high percentage of animals with only *C. perfringens* challenge exhibited marked intestinal lesion, include hyperemia, bleeding spots, thinner intestinal walls, and gas generation. According to Fig. 1a, the chickens with only *C. perfringens* challenge had the highest intestinal lesion score (*P* < 0.05), but dietary arginine supplementation significantly decreased intestinal lesion score in *C. perfringens* challenged birds (*P* < 0.05). The results of the histopathological evaluation were mainly in agreement with that of the gross lesion examination (Fig. 1b). As shown in Fig. 1c, the jejuna of birds in the CON group and the ARG group were almost normal, except for the presence of a few of lymphocytes. However, distinct epithelial cell defects, villus fusion, capillary hemorrhages, lymphocytes infiltration into the lamina propria and crypt abscesses were observed in most of the birds with only *C. perfringens* challenge. The degree of histopathological injury in ARG + CP group was significantly less than that in CON+CP group (*P* < 0.05). Figure 1d shows that there was a significant interaction effect between *C. perfringens* challenge and arginine addition on the number of *C. perfringens* in the liver (*P* < 0.05). No *C. perfringens* was detected in the livers of unchallenged birds. Challenged birds fed basal diets had a significantly higher number of *C. perfringens* in the liver than that in the unchallenged birds (*P* < 0.05). Challenged birds fed diets supplemented with arginine significantly reduced the liver *C. perfringens* number compared with the challenged birds fed basal diets (*P* < 0.05).

### Serum procalcitonin and arginine levels *in vivo*

**Fig. 2:**
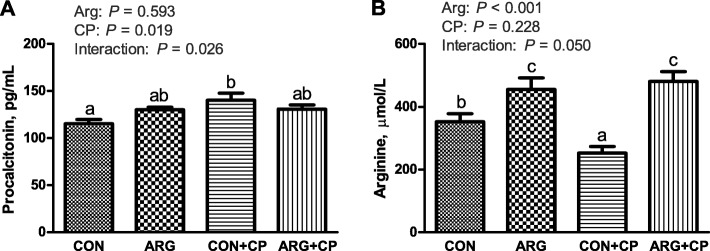
Serum procalcitonin (**a**) and arginine (**b**) concentrations of broiler chickens. CON group, received a basal diet; ARG group, fed a basal diet supplemented with 3 g/kg arginine; CON+CP group, received a basal diet and underwent *C. perfringens* challenge; ARG + CP group, given a basal diet supplemented with 3 g/kg arginine and underwent *C. perfringens* challenge. The results are expressed as means ± SEM (*n* = 8). Different letters indicate significant differences for the interaction effect (*P* ≤ 0.05)

Serum procalcitonin and arginine levels are shown in Fig. 2. *C. perfringens* challenge significantly increased the serum procalcitonin levels of birds (*P* < 0.05). A significant interaction was found between arginine supplementation and *C. perfringens* challenge on serum procalcitonin levels (*P* < 0.05). Compared with the CON group, the CON+CP group significantly increased the serum procalcitonin level (*P* < 0.05). However, arginine supplementation did not affect the serum procalcitonin level (*P* > 0.05). There was a significant interaction between arginine supplementation and *C. perfringens* challenge on the serum arginine levels (*P* = 0.05). The serum arginine level in the CP group was significantly lower than that in the CON group (*P* < 0.05), whereas chickens receiving the arginine supplemented-diet (ARG and ARG + CP groups) had significantly increased serum arginine levels (*P* < 0.05).

### Lysozyme and iNOS activities of jejunal mucosa *in vivo*

**Fig. 3:**
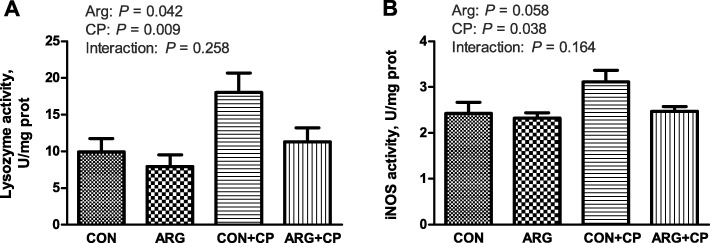
Jejunal mucosal lysozyme (**a**) and iNOS (**b**) activities of broiler chickens. CON group, received a basal diet; ARG group, fed a basal diet supplemented with 3 g/kg arginine; CON+CP group, received a basal diet and underwent *C. perfringens* challenge; ARG + CP group, given a basal diet supplemented with 3 g/kg arginine and underwent *C. perfringens* challenge. iNOS, inducible nitric oxide synthase. The results are expressed as means ± SEM (*n* = 8)

Significant increases in lysozyme and iNOS activities in the jejunal mucosa were observed after *C. perfringens* challenge (*P* < 0.05; Fig. 3). Compared with the effect of the basal diet, arginine supplementation significantly decreased lysozyme activity (*P* < 0.05) in the jejunal mucosa.

### Gene expression of jejunal inflammatory cytokines *in vivo*

**Fig. 4:**
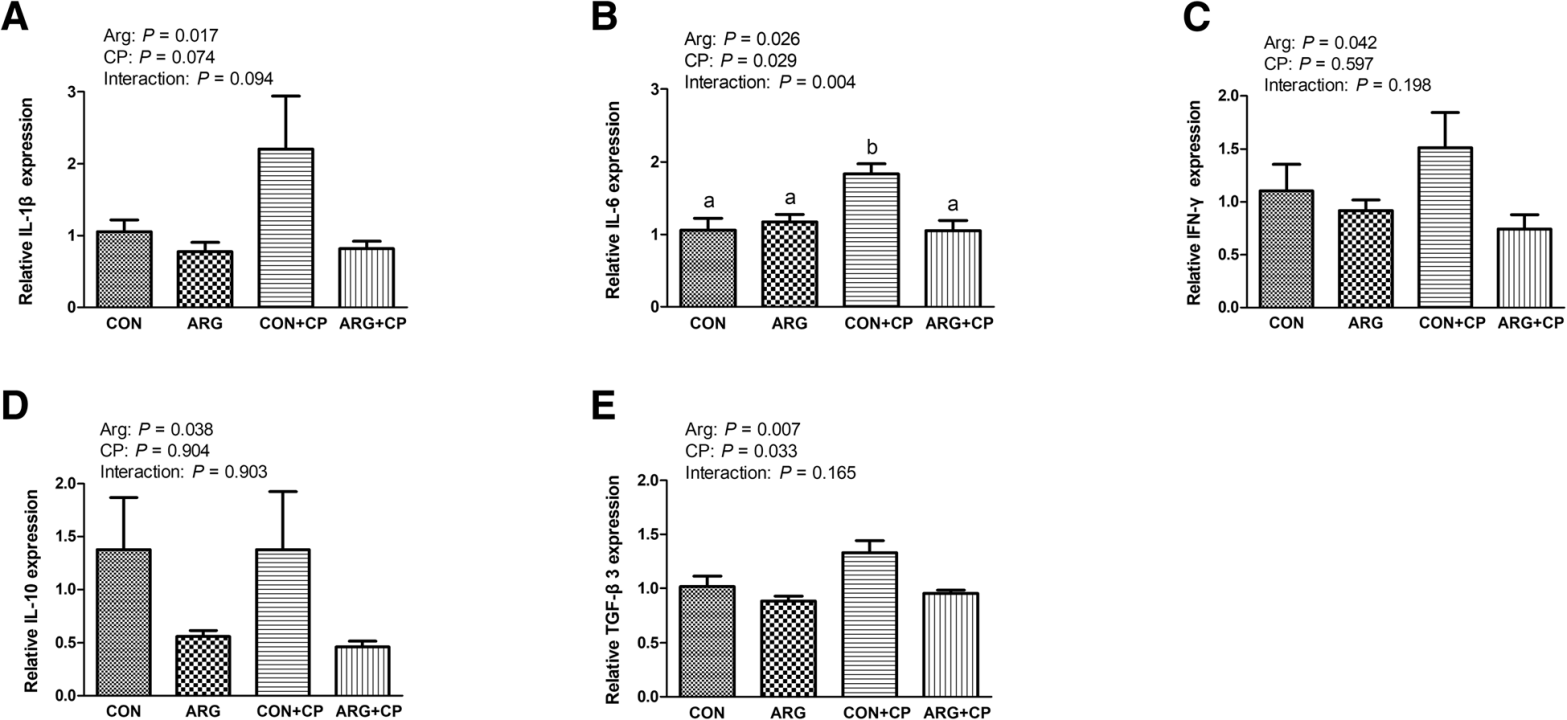
Gene expression of cytokines [*IL-1β* (**a**), *IL-6* (**b**), *IFN-γ* (**c**), *IL-10* (**d**) and *TGF-β3* (**e**)] in the jejunum of broiler chickens. CON group, received a basal diet; ARG group, fed a basal diet supplemented with 3 g/kg arginine; CON+CP group, received a basal diet and underwent *C. perfringens* challenge; ARG + CP group, given a basal diet supplemented with 3 g/kg arginine and underwent *C. perfringens* challenge. The results are expressed as means ± SEM (*n* = 8). Different letters indicate significant differences for the interaction effect (*P* ≤ 0.05)

As shown in Fig. 4, *C. perfringens* challenge significantly enhanced the mRNA expression of *IL-6* and *TGF-β3* (*P* < 0.05) in the jejunum. Arginine supplementation significantly reduced the mRNA expression of *IL-1β*, *IL-6*, *IFN-γ*, *IL-10*, and *TGF-β3* (*P* < 0.05) in the jejunum. Furthermore, the interaction between the effects of arginine supplementation and *C. perfringens* challenge dramatically influenced jejunal *IL-6* mRNA expression (*P* < 0.05). *IL-6* mRNA expression in the CP group was the highest among the four groups, and there were no significant differences among the other three groups.

### Gene expression of jejunal cationic amino acid transporters *in vivo*

As shown in Table 3, the challenged groups had higher *CAT-1* mRNA expression in the jejunum than did the unchallenged groups (*P* < 0.05). Arginine supplementation significantly suppressed jejunal *CAT-2* and *CAT-3* mRNA expression (*P* < 0.05). A significant interaction was found between the effects of arginine supplementation and *C. perfringens* challenge on jejunal *CAT-3* mRNA expression (*P* < 0.05). The birds with only *C. perfringens* challenge had the highest jejunal *CAT-3* mRNA expression, and arginine supplementation significantly reduced jejunal *CAT-3* mRNA expression in the challenged birds (*P* < 0.05).

**Table 3:** Gene expression of *CATs* and arginine catabolic enzymes in the jejunum of broiler chickens

Items^c^		*CAT* ^d^ *-1*	*CAT-2*	*CAT-3*	*iNOS* ^e^	*ARG2* ^f^	*ADC* ^g^	*AGAT* ^h^
CON		1.00	1.03	1.01^a^	1.02^a^	1.02	1.03^a^	1.01
ARG		1.05	0.64	0.98^a^	1.02^a^	0.79	0.93^a^	0.59
CON+CP		1.38	1.01	1.28^b^	1.73^b^	1.31	1.35^b^	1.03
ARG + CP		1.09	0.61	0.89^a^	0.93^a^	0.99	0.79^a^	0.92
SEM		0.049	0.063	0.047	0.087	0.067	0.066	0.056
Main effect								
Arg	–	1.21	1.02	1.15	1.38	1.16	1.19	1.02
	+	1.07	0.62	0.93	0.98	0.88	0.87	0.76
CP	–	1.03	0.84	0.99	1.02	0.92	0.98	0.80
	+	1.23	0.81	1.09	1.33	1.18	1.09	0.98
*P-*value								
Arg		0.169	0.001	0.008	0.003	0.028	0.006	0.008
CP		0.022	0.789	0.209	0.018	0.048	0.432	0.059
Interaction		0.060	0.994	0.023	0.003	0.661	0.041	0.089

^a,b^Different letters indicate significant differences for the interaction effect (*P* ≤ 0.05)

^c^CON group, received a basal diet; ARG group, fed a basal diet supplemented with 3 g/kg arginine; CON+CP group, received a basal diet and underwent *C. perfringens* challenge; ARG + CP group, given a basal diet supplemented with 3 g/kg arginine and underwent *C. perfringens* challenge

^d^*CAT* Cationic amino acid transporter

^e^*iNOS* Inducible nitric oxide synthase

^f^*ARG2* Arginase2

^g^*ADC* Arginine decarboxylase

^h^*AGAT* Arginine:glycine amidinotransferase

### Gene expression of the jejunal arginine catabolic enzymes *in vivo*

As shown in Table 3, *C. perfringens* infection significantly increased the mRNA expression of *iNOS* and arginase 2 (*ARG2*) (*P* < 0.05) in the jejunum of birds. Arginine supplementation significantly decreased the mRNA expression of *iNOS*, *ARG2*, *ADC*, and *AGAT* (*P* < 0.05). There were interactions between the effects of arginine supplementation and *C. perfringens* challenge on jejunal *iNOS* and *ADC* mRNA expression (*P* < 0.05). Compared with the CON and the ARG groups, the CP group had higher mRNA expression levels of *iNOS* and *ADC* in the jejunum (*P* < 0.05); however, the ARG + CP group significantly downregulated the mRNA expression for both of these enzymes compared with that in the CP group (*P* < 0.05).

### Gene expression of jejunal JAK-STAT signaling pathway components *in vivo*

**Fig. 5:**
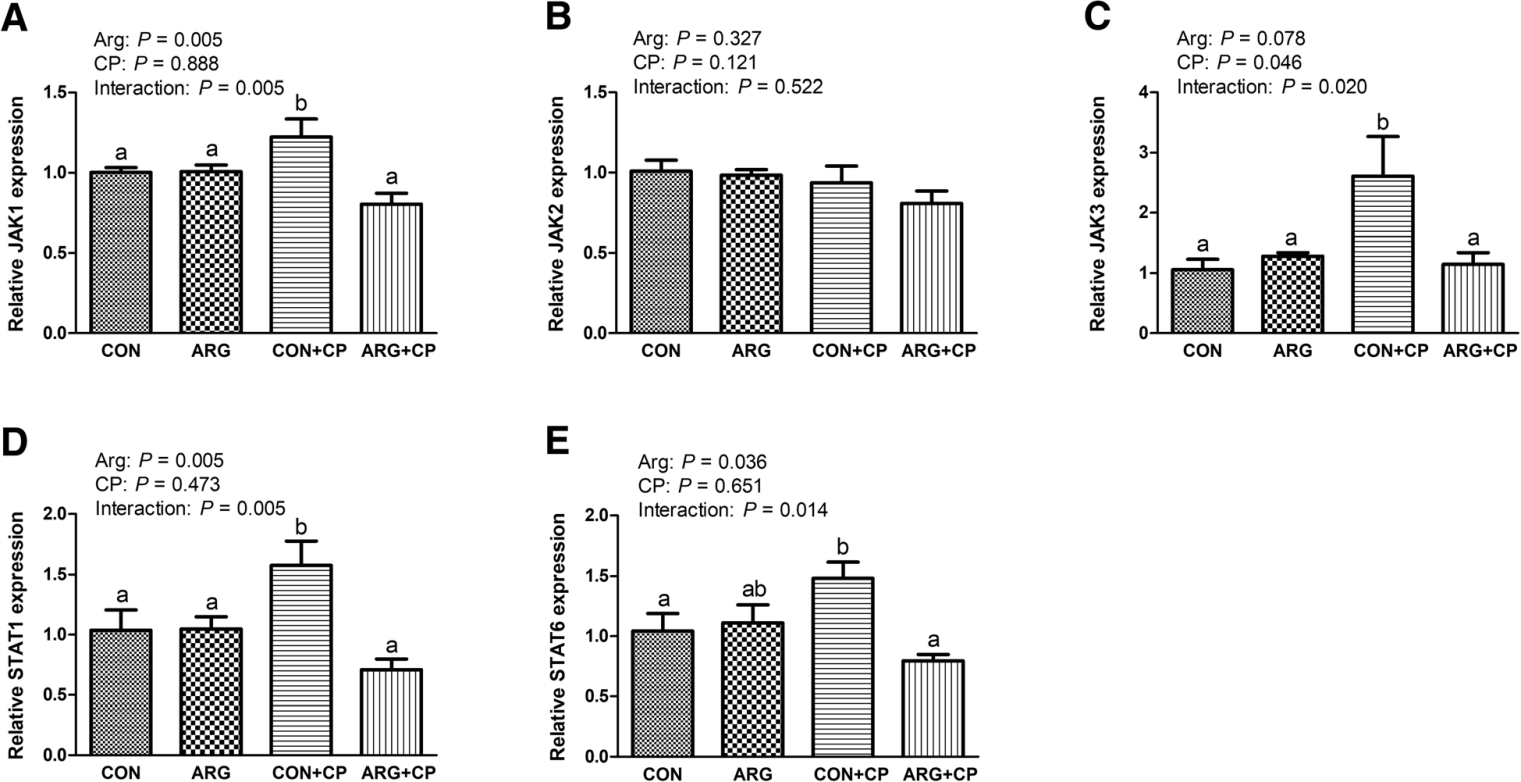
Gene expression of *JAK* [*JAK1* (**a**), *JAK2* (**b**) and *JAK3* (**c**)] and *STAT* [*STAT1* (**d**) and *STAT6* (**e**)] in the jejunum of broiler chickens. CON group, received a basal diet; ARG group, fed a basal diet supplemented with 3 g/kg arginine; CON+CP group, received a basal diet and underwent *C. perfringens* challenge; ARG + CP group, given a basal diet supplemented with 3 g/kg arginine and underwent *C. perfringens* challenge. *JAK*, Janus kinase; *STAT*, signal transducer and activator of transcription. The results are expressed as means ± SEM (*n* = 8). Different letters indicate significant differences for the interaction effect (*P* ≤ 0.05)

Figure 5 demonstrates that *C. perfringens* challenge induced a significant increase in jejunal *JAK3* mRNA expression (*P* < 0.05). Arginine supplementation led to a reduction in jejunal *JAK1*, *STAT1*, and *STAT6* mRNA expression (*P* < 0.05). The effects of arginine supplementation and *C. perfringens* challenge had a significant interaction on the mRNA expression of *JAK1*, *JAK3*, *STAT1*, and *STAT6* in the jejunum of broiler chickens (*P* < 0.05). Compared with the control group, birds treated with only *C. perfringens* challenge had markedly increased the gene expression of *JAK1*, *JAK3*, *STAT1*, and *STAT6* in the jejunum (*P* < 0.05), while arginine supplementation significantly reversed these elevated gene expressions in the jejunum of birds challenged with *C. perfringens* (*P* < 0.05).

### Cytotoxicity, inflammation responses and the mRNA expression of CATs *in vitro*

**Fig. 6:**
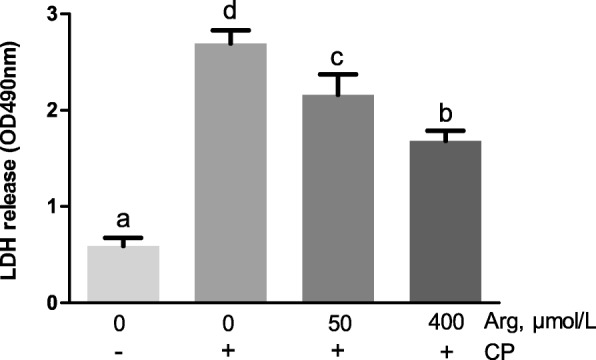
LDH release in supernatant of chicken intestinal epithelial cells stimulated with *C. perfringens*. Cells were cultured for 6 h in arginine-free DMEM/F12 containing 0, 50, 400 μmol/L arginine followed by incubating with or without *C. perfringens* for 4 h; LDH, Lactic dehydrogenase. The results are expressed as means ± SEM (*n* = 6). Bars sharing different lowercase letters differ markedly (*P* < 0.05)

**Fig. 7:**
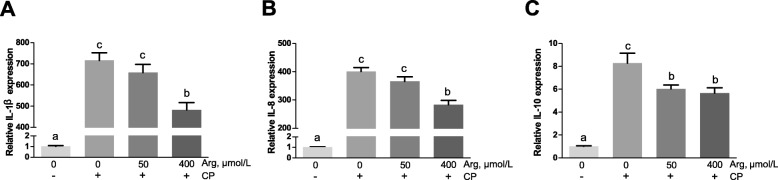
Gene expression of cytokines [*IL-1β* (**a**), *IL-8* (**b**) and *IL-10* (**c**)] in chicken intestinal epithelial cells stimulated with *C. perfringens*. Cells were cultured for 6 h in arginine-free DMEM/F12 containing 0, 50, 400 μmol/L arginine followed by incubating with or without *C. perfringens* for 4 h. The results are expressed as means ± SEM (*n* = 6). Bars sharing different lowercase letters differ markedly (*P* < 0.05)

**Fig. 8:**
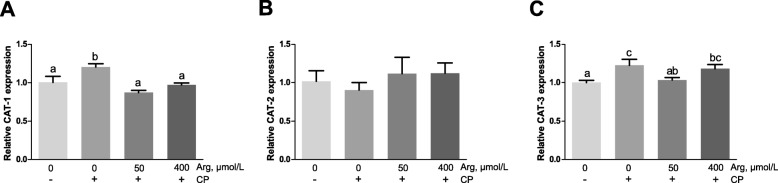
Gene expression of *CATs* [*CAT-1* (**a**), *CAT-2* (**b**) and *CAT-3* (**c**)] in chicken intestinal epithelial cells stimulated with *C. perfringens*. Cells were cultured for 6 h in arginine-free DMEM/F12 containing 0, 50, 400 μmol/L arginine followed by incubating with or without *C. perfringens* for 4 h. *CAT*, cationic amino acid transporter. The results are expressed as means ± SEM (*n* = 6). Bars sharing different lowercase letters differ markedly (*P* < 0.05)

Figure 6 shows that *C. perfringens* challenge caused a significant increase in the cytotoxicity when compared with the unchallenged chicken IEC (*P* < 0.001). However, pre-treatment with arginine (50 μmol/L and 400 μmol/L) prior *C. perfringens* challenge significantly decreased the cytotoxicity (*P* < 0.001) and the 400 μmol/L arginine group reduced the cytotoxicity more than the 50 μmol/L arginine group (*P* < 0.001). As revealed in Fig. 7, the mRNA abundances of *IL-1β*, *IL-8*, and *IL-10* in *C. perfringens*-challenged group (group 2) were 717.20-fold (*P* < 0.001), 400.59-fold (*P* < 0.001) and 8.29-fold (*P* < 0.001) higher than that in the unchallenged group, respectively. The 50 μmol/L arginine group could not affect *IL-1β* and *IL-8* mRNA expression but significantly decreased the *IL-10* mRNA expression (*P* < 0.001) when compared with *C. perfringens*-challenged group. The 400 μmol/L arginine group significantly reduced the increased mRNA abundances of *IL-1β*, *IL-8*, and *IL-10* caused by *C. perfringens* challenge (*P* < 0.001). Figure 8 exhibits that the mRNA expression of *CAT-1* (*P* < 0.001) and *CAT-3* (*P* < 0.05) was significantly stimulated in response to *C. perfringens* challenge. The increased mRNA expression of *CAT-1* was markedly decreased by 50 μmol/L and 400 μmol/L arginine pre-treatment (*P* < 0.001). Furthermore, 50 μmol/L arginine group significantly decreased the increased *CAT-3* mRNA expression induced by *C. perfringens* challenge (*P* < 0.05) while 400 μmol/L arginine group did not affect *CAT-3* mRNA expression compared with *C. perfringens*-challenged group (*P* > 0.05).

## Discussion

Gross lesion score and histopathological examination are commonly used for evaluating the severity of necrotic enteritis. In this study, increased intestinal lesion and histopathological injury scores were observed in *C. perfringens* challenged chickens relative to the control group, suggesting that *C. perfringens* infections led to inflammatory responses in the intestine. As each group of birds was kept in a rearing isolator and there was no replicate, so it is a pity that we did not determine the growth performance. For the *C. perfringens*-infected birds, arginine addition markedly decreased the intestinal gross pathological and histopathological lesion scores compared with the control diet group, indicating that arginine benefited the intestinal health. In addition, *C. perfringens* challenged birds exhibited higher levels of *C. perfringens* in the liver compared with the unchallenged birds, reflecting that *C. perfringens* challenge damaged gut barrier integrity. However, dietary arginine supplementation prevented the invasion of *C. perfringens* into the liver, suggesting that arginine addition played a positive role in controlling *C. perfringens* challenge*.* The *in vitro* results demonstrated that arginine pre-treatment attenuated the *C. perfringens*-induced amounts of LDH, an indicator of cytotoxicity. In line with these results, our previous studies have reported that arginine supplementation could promote intestinal health, as indicated by improving intestinal absorption function, decreasing intestinal permeability and inhibiting intestinal *C. perfringens* population in the broilers under the conditions of subclinical necrotic enteritis [19].

Procalcitonin serves as a biomarker of severe bacterial infections [28]. In bacterial infections, bacterial endotoxins and inflammatory cytokines stimulate procalcitonin production in many tissues, such as liver, lung and kidney, and then, procalcitonin goes in circulation, leading to a marked increase in the serum procalcitonin concentration [29]. In our study, increased serum procalcitonin levels after *C. perfringens* challenge indicated a systemic inflammatory response, which also evidenced that the infectious model was successfully established.

Arginine availability in the digestive tract plays a key role in maintaining intestinal immune homeostasis under conditions of inflammation and infection [6]. Total body arginine content has been found to be depleted in response to trauma or surgery [30]. However, whether *C. perfringens* infection induces a systemic arginine deficiency in broiler chickens remains unclear. In this study, *C. perfringens*-infected birds fed basal diets significantly reduced serum arginine levels compared to the uninfected birds fed basal diets, which indicated that an arginine deficiency developed in response to *C. perfringens* infection. Arginine administration has been shown to partially or completely reverse arginine deficiency-associated immunopathologies, ameliorating conditions such as intestinal ischemia, malabsorption, inflammation and infectious complications [6, 11, 13], which supports our results.

In addition to nutrient digestion and absorption, the intestine also plays a key role in the immune response. Lysozyme, an important bacteriolytic component of the innate immune system, can hydrolyze glycosidic bonds in the peptidoglycan layer of Gram-positive bacterial cell walls [31]. Moreover, lysozyme inhibited the growth of *C. perfringens* and alpha toxin production [32]. Anaerobic cow mastitis induced by *C. perfringens* increased lysozyme activity in milk samples, which was positively correlated with disease severity [33]. Our results revealed that the elevated lysozyme activity in the jejunal mucosa caused by *C. perfringens* was reduced by arginine supplementation, likely because arginine alleviated the intestinal damage caused by the infection.

Cytokines are usually considered as markers of injury or infection [34]. Excessive production of pro-inflammatory cytokines may damage intestinal integrity and barrier function [35]. In the present study, *C. perfringens* challenge significantly increased the mRNA expression of pro-inflammatory cytokines in the jejunum of birds, which were decreased by arginine addition, indicating that arginine alleviated the excessive activation of inflammatory response. Similar results were also reported in many other stress models, such as burn [36], sepsis [37] and gut ischemia-reperfusion [38]. Besides, the *in vivo* study illustrated that arginine administration down-regulated the increased transcription of anti-inflammatory cytokines induced by *C. perfringens* challenge. Tan et al. [39] reported that arginine addition could attenuate the increased transcription of both pro- and anti-inflammatory cytokines induced by lipopolysaccharide challenge in broiler chickens. In this *in vitro* study, the elevation of pro-inflammatory cytokines (*IL-1β*, 717.20 fold; *IL-8*, 400.59 fold) was much higher than that of anti-inflammatory cytokine (*IL-10*, 8.29 fold) when compared the only challenged cells with the unchallenged cells, indicating that pro-inflammatory response was dominant. However, arginine addition sharply decreased the elevation of *IL-1β* and *IL-8* mRNA expression induced by *C. perfringens* challenge. These results suggested that the gut injury was reversed by arginine addition through suppression of pro-inflammatory cytokines overproduction.

The intestine is an essential site for maintaining body arginine homeostasis [7]. Arginine is actively metabolized in enterocytes, and arginine transmembrane transport is mainly performed by the CAT family of proteins, which act through a Na-independent transport system [8]. The CAT family contains four members, CAT-1, CAT-2, CAT-3 and CAT-4, in which CAT-1, CAT-2, and CAT-3 transport cationic amino acids such as arginine, ornithine, and lysine [40], while CAT-4 is reportedly unable to transport basic, neutral or acidic amino acids, and its physiological function remains unknown [41]. The present study showed that *C. perfringens* challenge increased *CAT-1* and *CAT-3* mRNA expression in chickens and IEC, and arginine treatment reduced *CAT-2* and *CAT-3* mRNA expression in the jejunum of chickens, and *CAT-1* and *CAT-3* mRNA expression in IEC. Similarly, Zheng et al. [42] showed that dietary arginine supplementation reversed the increased mRNA expression of *CAT-1* induced by oxidative stress in the jejunum of weaned piglets. There are at least two factors in modulating arginine absorption. First, *CAT* mRNA content is regulated by substrate availability. Bogle et al. [43] found that arginine deprivation increased cationic amino acid transport in porcine aortic endothelial cells. This elevation may reflect adaptive responses in the CAT system. When arginine is sufficient, the expression of *CAT*s is less [42]. Second, CATs expression was modulated by IL-1β and IFN-γ. Previous studies have shown that IL-1β and IFN-γ stimulate arginine uptake, along with the Mrna expression of *CAT-1* and *CAT-2* in rat cardiac microvascular endothelial cells [44]. In this study, arginine supplementation downregulated the transcription of *IL-1β* and *IFN-γ*, as well as that of *CAT-2* *in vivo*, and suppressed the increased transcription of *IL-1β* and *CAT-1* induced by *C. perfringens* challenge *in vitro*, which evidenced that *IL-1β* and *IFN-γ* were involved in the regulation of *CATs* expression in chickens under *C. perfringens* challenge.

*L*-arginine is catabolized via multiple pathways mediated by iNOS, arginase, AGAT, and ADC, giving rise to nitric oxide, polyamines, proline, glutamate, creatine, and agmatine, as well as other products [9]. In our study, *C. perfringens* infection increased jejunal mRNA expression of *iNOS*, *ARG2* and *ADC*, as well as increasing iNOS activity in the jejunal mucosa, which stimulated arginine catabolism, leading to serum arginine deficiency. Dietary arginine supplementation normalized the overexpression of arginine catabolic enzymes, contributing to the maintenance of arginine homeostasis. But out of our expectation, the addition of arginine, the sole substrate of NO in body, did not increase the mRNA and protein level of iNOS but conversely decreased them in the *C. perfringens*-challenged birds. Similar findings were also reported by Zhao et al. [45] and Wu et al. [46]. The reasons why arginine decreased the *C. perfringens*-induced *iNOS* mRNA expression and activities may be as follows. First, iNOS level and NO synthesis vary over time. Kosaka et al. [47] discovered that arginine supplementation counteracted the decrease in arginine levels and increased NO in the kidney in the earliest phase (45 min) of ischemia-reperfusion in rats, however, it down-regulated the increased plasma NO as well as renal *iNOS* mRNA and protein expression at 7 h of ischemia-reperfusion. In our study, arginine administration may promote NO synthesis in the earliest phase of *C. perfringens* challenge, which then in turn inhibited excessive expression of *iNOS* gene [48] in order to avoid damages caused by excessive NO. Second, arginine may be preferentially catalyzed to ornithine by ARG 1. There are two forms of arginase: the cytoplasmic type ARG1 and the mitochondrial type ARG2 [11]. Arginase competes with iNOS for the common substrate, l-arginine. However, we did not determine *ARG1* mRNA expression in our study because the gene sequence of *ARG1* in chickens is not uncovered. Agmatine produced by ADC has no deleterious effects on bacteria and can be effectively used as an energy source by the pathogen [49]. Recently, an *in vitro* experiment showed that lipopolysaccharide (LPS) plus cytokines (IL-1β, IFN-γ and TNF-α) could upregulate the arginine decarboxylase pathway in astrocytes and the macrophage-like cell line RAW 264.7, resulting in increased agmatine production [50]. AGAT catalyzes the rate-limiting step of creatine production, and there are few studies on the role of AGAT in intestinal inflammation. Recently, creatine was found to protect against colitis in homozygous AGAT mutant mice [51]. In our study, the result that arginine administration decreased the mRNA expression of *iNOS*, *ARG2*, *ADC* and *AGAT* in the jejunum of chickens suggested that arginine alleviated the inflammatory injury after *C. perfringens* challenge-induced acute inflammation.

The JAK-STAT cascade is a critical signaling pathway in the inflammatory responses [15]. In the canonical JAK-STAT pathway, LPS or cytokine binding to its receptors induces JAK phosphorylation, which in turn phosphorylates STATs. Then, phosphorylated STATs form homodimers or heterodimers and translocate to the nucleus to regulate target gene transcription, including genes of other pro-inflammatory cytokines, chemokines, and inducible enzymes such as iNOS [16, 17]. Due to the deficiency of appropriate antibodies available for use in studies of chickens, we did not determine the protein levels and phosphorylation status of components in this signaling pathway. In the present study, the infected chickens increased jejunal *JAK1*, *JAK3*, *STAT1* and *STAT6* mRNA expression compared to uninfected birds. Consistently, some studies have reported that the mRNA levels of JAK-STAT pathway were changed after pathogen challenge. Truong et al. [18] demonstrated that mRNA expression of *JAK1*, *JAK3*, and *STAT1* was significantly increased in the spleen of broilers suffering from necrotic enteritis as compared with that of healthy birds. Besides, according to the RNA-Seq data, 29 and 19 differentially expressed genes were mapped to JAK-STAT signaling pathways in the spleens of Marek’s disease resistant chicken line and susceptible line co-infected with *Eimeria maxima*/*C. perfringens* compared to control, respectively [52]. In this study, arginine administration inhibited the *C. perfringens* challenge-induced gene expressions involved in the JAK-STAT pathway. Dörpinghaus et al. [53] reported that the inhibition of STAT1 expression downregulated *iNOS* transcription, which can alleviate LPS-induced cytotoxicity in macrophages and microglial cells. Ashino et al. [54] have proven that the selective inhibition of JAK1/3 reduced STAT6 and STAT5 phosphorylation and Th2 cytokine production, which suppressed the development of airway hyperresponsiveness. The results of our study revealed that the downregulation of activated JAK-STAT pathways was a possible mechanism by which arginine attenuated *C. perfringens*-induced gene expression of iNOS and some pro-inflammatory cytokines. In the present study, the changes of *STATs* at mRNA level may be related with the non-canonical pathway involving unphosphorylated STATs. Recently, some researchers uncovered that the unphosphorylated STAT3 could compete with IκB for binding to unphosphorylated NF-κB, translocating to the nucleus and participating in the activation of a subset of NF-κB-dependent genes [55]. Unphosphorylated STAT6 was suggested to bind to a consensus STAT6 binding site in the promoter of the *COX*-2 gene, regulating its constitutive expression [55].

## Conclusions

Dietary l-arginine supplementation prevented the circulating arginine deficiency induced by *C. perfringens* challenge and reversed *C. perfringens* challenge-upregulated gene expression of inflammatory cytokines, arginine transporters and arginine catabolic enzymes in the intestine of broiler chickens. The attenuation of the inflammatory response by arginine was at least partly via inhibition of JAK-STAT signalling pathways. These results indicate that arginine supplementation can be used as a potential preventive approach against avian necrotic enteritis.

## Data Availability

All data generated or analysed during this study are available from the corresponding author by request. The datasets supporting the conclusions of this article are included in the article.

## Abbreviations

ADC: Arginine decarboxylase
AGAT: Arginine:glycine amidinotransferase
ARG + CP: Birds received a basal diet supplemented with 3 g/kg arginine and *C. perfringens* challenge
ARG: Birds received a basal diet supplemented with 3 g/kg arginine
ARG2: Arginase 2
CAT: Cationic amino acid transporter
CON: Birds received a basal diet
CON+CP: birds received a basal diet and *C. perfringens* challenge
DMEM/F12: Dulbecco’s Modified Eagles Medium/Nutrient Mixture F-12
HPLC: High-performance liquid chromatography
IEC: Intestinal epithelial cells
iNOS: Inducible nitric oxide synthase
JAK: Janus kinase
LDH: Lactic dehydrogenase
MOI: Multiplicity of infection
PBS: Phosphate-buffered saline
STAT: Signal transducer and activator of transcription
